# Machine learning phenomics (MLP) combining deep learning with time-lapse-microscopy for monitoring colorectal adenocarcinoma cells gene expression and drug-response

**DOI:** 10.1038/s41598-022-12364-5

**Published:** 2022-05-20

**Authors:** M. D’Orazio, M. Murdocca, A. Mencattini, P. Casti, J. Filippi, G. Antonelli, D. Di Giuseppe, M. C. Comes, C. Di Natale, F. Sangiuolo, E. Martinelli

**Affiliations:** 1 Department of Electronic Engineering, University of Rome Tor Vergata, via del Politecnico 1, 00133 Rome, Italy; 2 Interdisciplinary Center for Advanced Studies on Lab-on-Chip and Organ-on-Chip Applications (ICLOC), University of Rome Tor Vergata, 00133 Rome, Italy; 3 Department of Biomedicine and Prevention, Tor Vergata University, Rome, Italy

**Keywords:** Machine learning, Biomedical engineering, Gene expression

## Abstract

High-throughput phenotyping is becoming increasingly available thanks to analytical and bioinformatics approaches that enable the use of very high-dimensional data and to the availability of dynamic models that link phenomena across levels: from genes to cells, from cells to organs, and through the whole organism. The combination of phenomics, deep learning, and machine learning represents a strong potential for the phenotypical investigation, leading the way to a more embracing approach, called machine learning phenomics (MLP). In particular, in this work we present a novel MLP platform for phenomics investigation of cancer-cells response to therapy, exploiting and combining the potential of time-lapse microscopy for cell behavior data acquisition and robust deep learning software architectures for the latent phenotypes extraction. A two-step proof of concepts is designed. First, we demonstrate a strict correlation among gene expression and cell phenotype with the aim to identify new biomarkers and targets for tailored therapy in human colorectal cancer onset and progression. Experiments were conducted on human colorectal adenocarcinoma cells (DLD-1) and their profile was compared with an isogenic line in which the expression of LOX-1 transcript was knocked down. In addition, we also evaluate the phenotypic impact of the administration of different doses of an antineoplastic drug over DLD-1 cells. Under the omics paradigm, proteomics results are used to confirm the findings of the experiments.

## Introduction

A major inhibition to successful cancer treatment is the widespread heterogeneity in tumour cell populations, at the patient as well as at the cell level^1^. Cancer cells vary in their response to therapy, in the development of drug tolerance, survival and metastatic potential. The evolution of multidrug-resistant genotype has been noted in subsets of hematologic and solid tumours including breast, ovarian, lung, and lower gastrointestinal tract cancers^1^. Such heterogeneity explains why personalized therapies based on tumours molecular phenotyping have acquired great interest, with the final aim to perform genome disease stratification and identify specific treatments for each detected disease subcategory^2^. However, the great spatio-temporal complexity of the molecular phenomena, the gene redundancy and pathways functional overlaps, makes it arduous identifying the inter-relationships among specific genetic/epigenetic traits and cancer phenotypes^3,4^.

Moving from a large-scale population genomics investigation to a focused phenomics approach, it is then possible to identify physiologically relevant measures of disease risk using a smaller subset of patients and to derive clinically meaningful and translational insights on disease aetiology^5,6^. Such an approach is particularly powerful to account for potential modifiers of disease risk (e.g., microenvironment, microbiome, family history, longitudinal follow-up, etc.). Although analyses of genomic data have been successful at uncovering biological phenomena, they are—in most cases—supplementing rather than supplanting phenotypic information^7^. Phenotyping investigation (*phenomics* in what follows) is therefore crucial to address exceptional responders in cancer realm or rare diseases^8^.

**Figure 1:**
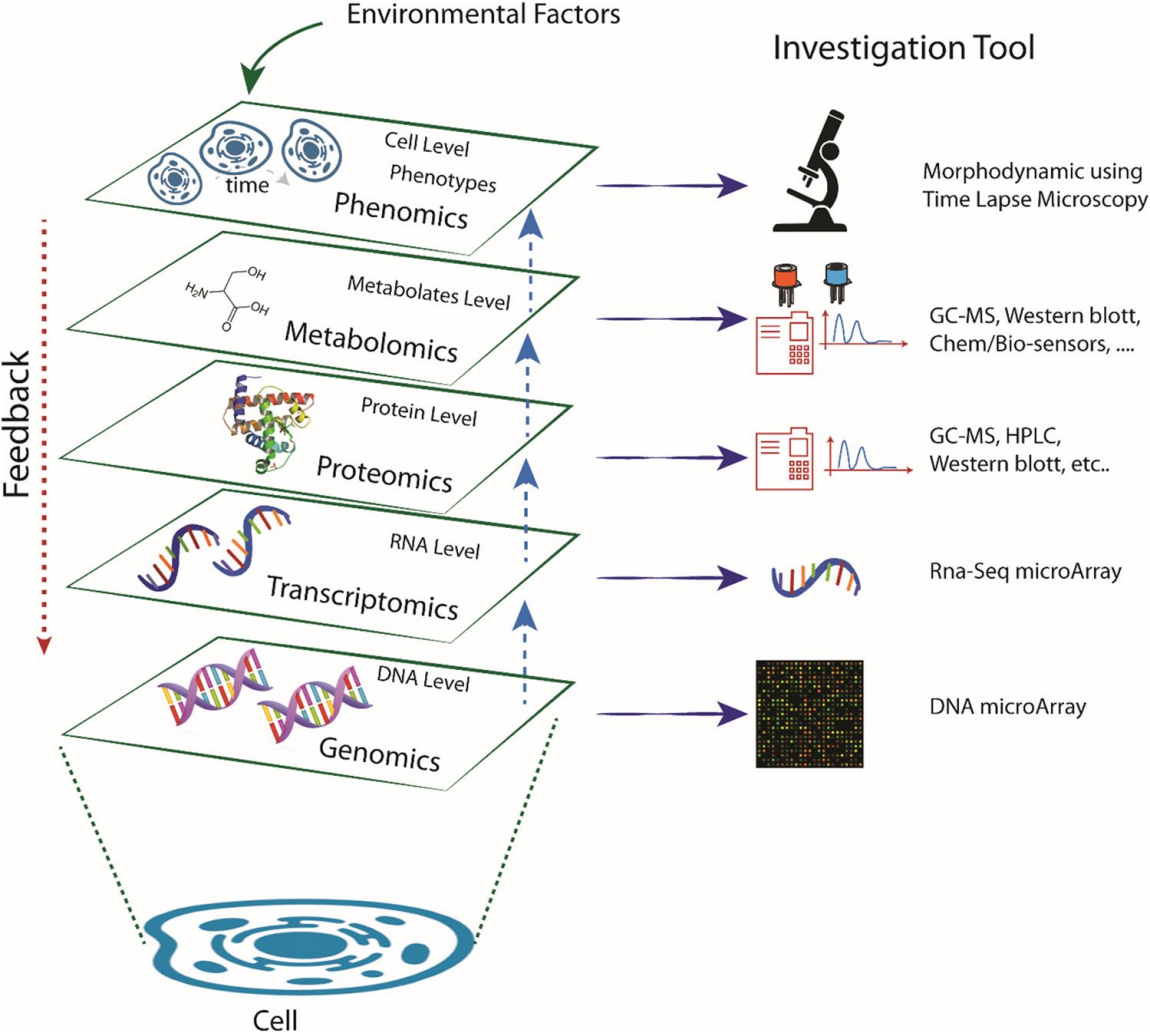
A layout of the genomics-phenomics axis. The cell behavior may be investigated at different scales: from the DNA microarray analysis for genomics, through proteomics and metabolomics by gas chromatography mass spectrometry, until a microscale analysis for phenomics using optical imaging acquisition.

In the present work, we claim to integrate phenotype disease manifestations (so called *observational traits*) with physiologic, multi-omic, and imaging data^9,10^ (see Fig. 1). Challenging is therefore to quantitatively understand phenotypic characteristics, such as health, disease and evolutionary behaviour of cells, organs, and individuals at small-scale levels^11^. Phenotypic variation is produced through a complex set of interactions between genotype and environment and such a ‘Genotype–Phenotype’ (GP) map is inaccessible without the detailed phenotypic data that allow these interactions to be studied. Fortunately, time is ripe for phenomics paradigm application: technologies for high-throughput phenotyping are becoming increasingly available^12^; analytical and bioinformatics approaches that enable the use of very high-dimensional data are advancing rapidly^13^; dynamic models that link phenomena across levels from genes to cells, to organs and through the whole organism are in reach. Figure 1 represents some investigation tools nowadays available for each omic scenario, going from DNA and RNA-Seq microarray for genotype investigation to gas chromatography mass spectrometry (GC–MS) for proteomics/metabolomics and finally, at larger scale, morphodynamic (phenotype) analysis through time-lapse microscopy.

The common denominator of cancer cell heterogeneity to drug response relies on the cell-to-cell variability even between genetically identical cells^14^, caused by epigenetic differences, cellular microenvironment, differences in protein expression, and asymmetric cell division^15,16^. This variability has been shown to influence and determine cellular decision-making and has been studied in various contexts, including cancer drug resistance^14^, pluripotency of stem cells^17^, and microbial infection^18^. Relevantly, studies have indicated that biological systems have evolved to exploit cell-to-cell variability in a sort of survival/adaptation task^19,20^.

To reveal variability in cellular signalling and exploit it to the aim of disease reaction understanding and control, measurements at the single-cell level^21^ (generally indicating the fact that phenotype information are extracted from each cell along time) as well as at a subgroups of cells are required^22^.

Existing experimental techniques comprise live-cell imaging using fluorescent markers^23^ or mass^24^ and flow cytometry^25^, and fluorescence label-free time-lapse microscopy^26,27^. The data provide different types of information about the cells, going from kinematics characteristics of individual cells trajectories to cell morphometric states classification^20^.

Phenomics may enlarge the plethora of cell analyses by suggesting more and more aspects of cell dynamics to be investigated. Cell dynamic characteristics (kinematics, morphodynamics exploited along each cell trajectory) contain several coded information useful to extract not only individual cell activities information but also the likelihood of entire population behaviour. On the other hand, the complexity of such phenotypical information may confound the extraction of the so-called *latent factors* for the final understanding of the population drug reaction mechanism (resistance, efficacy, abnormal response), to the ultimate task of targeting and optimize the treatment for a subclass of individuals.

Keeping this in mind, in this work we present a novel platform for phenomics investigation of cancer cells response to therapy, exploiting and combining the potential of time-lapse microscopy for cell behaviour data-acquisition and robust deep learning software architectures for the latent phenotypes extraction^28^.

A two-steps proof of concepts is designed to illustrate some of the possible phenomics insights revealed by the proposed platform.

First, we demonstrate a strict correlation among gene expression and cell phenotype conducting experiments on the human colorectal adenocarcinoma cells (DLD-1) and comparing its profile with an isogenic line in which the expression of LOX-1 transcript is down modulated by using siRNA approach^29^. The identification of new biomarkers and targets for tailored therapy in human colorectal cancer (CRC) onset and progression is an interesting challenge. CRC tissue produces an excess of ox-LDL, suggesting a close correlation between lipid dysfunction and malignant transformation. Lectin-like oxidized LDL receptor-1 (LOX-1) is involved in several mechanisms closely linked to tumorigenesis^30–32^. In our previous publications^29,33^, we designed and built a cell line in which in vitro expression of LOX-1 is stably knockdown by siRNA and we called them LOX- 1RNA_i_ DLD-1. Specifically, we used a shRNA-expressing lentiviral vector targeting the mRNA encoded by the orl1 gene. In these cells the modulation of LOX-1 leads to an impaired proliferation rate, affecting cell growth and tumorigenicity. Thus, in this work we do not have a mutated versus a wild type cells, but the in vitro experiments just compare DLD-1 versus LOX- 1RNA_i_ DLD-1 having an identical genetic background, except for LOX-1 expression. Moreover, a drug was used able to inhibit VEGF, also demonstrating that VEGF and LOX-1 are mutually regulated.

This fact motivated the interest in this case study as a proof of concept of the existence and the importance of the genomics-phenomics relationship. In addition, we demonstrated the existence of a proteomics-phenomics (PP) axis by administering over DLD-1 cells different doses of Bevacizumab, a well-known antineoplastic drug. Such a drug is a Vascular Epithelial Grow Factor (VEGF) specific antibody. Bevacizumab selectively binds VEGF preventing it from binding its receptors. There is a strict correlation between LOX-1 and VEGF: when overexpressed LOX-1 determine the upregulation of VEGF expression inducing the neoangiogenic process in many tumours^34,35^. VEGF targeted therapies, such as bevacizumab, decrease the concentration of VEGF hence exerting effects on vasculature, inhibiting new vessel growth and regrading newly formed vessels, as well as directly on cells (cytostatic effect)^36–38^.

In the two aforementioned studies, we depict biological scenarios in which by varying the expression level of a single transcript (LOX-1 and VEGF) within the same genetic background we tried to measure the corresponding phenotypic variation, reconstituting the bridge of the GP and of the PP association. The two case studies are interconnected since LOX-1 and VEGF are tightly and mutually linked.

These preliminary case studies are also used as a proof of concept of the possibility to recreate the GP and the PP association from phenomics in the simpler environment of the 2D culturing examination. Under the omics paradigm, proteomics results are used to confirm the findings of the experiments. Notably, we found that cells with similar expression of LOX-1 and VEGF share the same observable phenotype.

The combination of phenomics, deep learning, and machine learning represents a strong potential for phenotypical investigation overcoming the need to define hand-crafted descriptors, although highly meaningful, leading the way to a more embracing phenomics approach, called *Machine Learning Phenomics* (MLP). MLP avoids the need to define specific descriptors focusing the analysis on a detailed aspects (e.g., cell kinematics, cell death events, etc.) but rather to extract a high-level time-varying phenotypical representation, at single-cell level, for the duration of the experiment. Such a high-level encoding, endorsed by transfer learning procedure^39,40^, allows solving the curse of dimensionality problem and reaching a strong generalizability ability. At the same time, the proposed MLP platform introduces, for the first time, the possibility to conduct continuous phenotypical investigation along time, revealing not only gene and drug-related cell state fate but also the signalling pathway along the response processes. This aspect is, to author’s opinion, crucial for a further advance in cell phenomics investigation, featuring the possibility to redefine personalized drug treatment protocols not only in concentration and cocktail formulation, but also in relation to *drug timing*. Drug dosing and timing are still partially undiscovered aspects that may play a crucial role in pharmacokinetics paradigms toward a more precise understanding of drug resistance phenomena and abnormal drug responses mechanism, with the aim to optimize the cancer treatment long-term effects, also in relation to the genotype^41^.

## Results

### Experimental set-up

We evaluated our approach on two different studies:

- **Case study 1.** The phenomics representation evidenced comparing colorectal adenocarcinoma DLD-1 cells versus the same cell line in which only LOX-1 mRNA expression has been in vitro downmodulated by a siRNA approach.
- **Case study 2.** The recognition of phenotype patterns of dose-mediated effects of drug administered to DLD-1 cells (Bevacizumab at two different concentrations, 125 µg/ml and 250 µg/ml), versus DLD-1 in which no drug has been added.

For both scenarios, phenotype responses are collected by using deep transfer learning, namely by using pretrained deep learning networks with the aim to convert images (region of interest containing a cell along its trajectory) into numerical descriptors. The variation of the descriptors along the trajectory is then quantified by computing the standard deviation of the descriptors over time. In this way, the method is automatically capable to characterize phenotype variations over time and use them to construct a MLP model for the task of classification. We compared our approach against another benchmark method involving the construction of a classification model over standard morphological phenotypical descriptors (perimeter, area, eccentricity and solidity of cell shape) and cell texture features (Haralick features^42^). Additional comparative approaches will be also presented with the aim to demonstrate the effectiveness of the algorithm blocks designed (e.g., background subtraction, the role of deep features with respect to standard object segmentation, etc.) in relation to an increased algorithm robustness to luminance variations for example. Experiments were conducted in a 2D microenvironment using label-free time lapse microscopy in the brightfield. Video are acquired at a frame rate of 1 frame per minute and at a spatial resolution of 0.33 μm per pixels. The total duration of each experiment was 12 h.

### Experimental results

#### Case study 1

##### Performance of classification

In case study 1, we were involved in the task of discriminating phenotype variations in DLD-1 cells with respect to DLD-1 in which LOX-1 expression has been reduced (later simply LOX1 inhibited), demonstrating the existence of a strict correlation among gene expression and cell phenotype in this biological system. In the considered experiment, cells naturally tended to group in clusters. We postulated that cells of the same cluster behad in a sort of cooperative way that may be revealed by computing the majority voting over the class labels assigned to the tracks of the same cluster. Clusters are automatically identified by implementing the procedures described in^43^. In particular, to demonstrate such an assumption, we compare the results achieved by the Support Vector Machine (SVM)^44^ classification model, built over descriptors of each single track, with those achieved using voting at the cluster level. Further details of the classification models settings can be found in the Method section.

Results have been evaluated by computing the unbalanced accuracy of classification calculated on half-experiment-out cross-validation. We divided each video into two identical partitions (left and right). Clusters belonging to one of the two partitions were used in test in each running whereas the remaining partitions were used as training set. For this reason, we referred to this kind of cross-validation as “half-experiment out”.

In order to demonstrate the generalizability of the approach, we compare the accuracy results obtained by using the four different networks: AlexNET^45^, GoogleNET^46^, ResNET101^47^, and NasNETLarge^48^. Table 1 lists the accuracy values.

**Table 1:** Accuracy values of classification using four deep learning network and SVM classification model.

DLD-1 versus LOX1 inhibited	ALEXNET	GOOGLENET	RESNET101	NASNETLARGE
**Single-track level**	72.83 ± 5.22%	71.55 ± 4.85%	74.00 ± 4.37%	74.44 ± 4.40%
**Single-cluster level**	70.00 ± 7.73%	79.52 ± 7.48%	80.48 ± 6.59%	82.38 ± 5.66%

**Figure 2:**
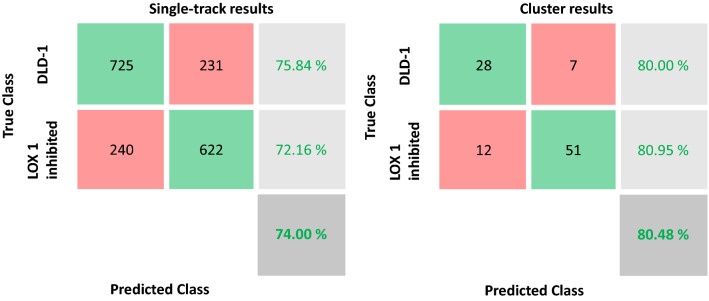
Confusion matrices of the classification task in case study 1 using RESNET101 and SVM classifier. Single-track results (left) and cluster results (right). Third column represents in turn from top to bottom: ratio of DLD1 correctly recognized, ratio of LOX1 inhibited correctly detected, unbalanced accuracy (ratio of the sum of true positives and true negatives over the total number of instances).

It is important to observe that the four networks obtain almost the same results, with a maximum variation in the mean accuracy value of 2.89%, at single-track level. Such an invariance gives strength to the approach and demonstrates its generalizability. In light of this, more specific results in terms of confusion matrices can be found in Fig. 2 only for RESNET101 network and the SVM classifier.

As it can be seen, out of 35 clusters (956 tracks) with DLD-1 cells, 28 are recognized as DLD-1 whilst only 7 are as assigned to LOX1 inhibited. On the contrary, out of the 63 clusters belonging to LOX1 inhibited (862 single tracks), 51 are recognized as LOX1 inhibited whilst only 12 are assigned to DLD-1 class.

##### Comparison with traditional morphological features

In order to appreciate the fundamental role of the dynamic features extracted and to demonstrate their discrimination ability in terms of classification accuracies, we perform a comparative test: in particular, we considered the results obtained by constructing the classification model over the standard morphological descriptors (perimeter, area, eccentricity and solidity of cell shape) and the cell texture features (Haralick features^42^), mentioned in the Introduction. All the extracted descriptors are coded by computing five statistics over the trajectory: mean, standard deviation, skewness, kurtosis and signal Shannon Entropy. In order to extract such morphological descriptors, we needed to segment cells in each ROI. Segmentation was carried out using supervised approach based on semantic segmentation (SS) by Deeplab v3 + architecture^49^. Such architecture used a pretrained CNN network, RESNET101, and performed retraining of the labelling step using a given amount of images with assigned ground truth segmentation. The augmentation technique was used to enlarge the dataset for training, by shifting and rotation. In this way, SS can be adapted to whichever number of output levels (more than two) in presence of multi-labels images. In our application, we trained SS on a binary problem of segmenting cells over a dark background. After each cell shape is estimated, morphological parameters are extracted along with texture parameters computed in the cell region. Such features are then used to construct a unique classification model.

With the aim to compare results obtained by different classification model we compare here four distinct well-known classifiers: Support Vector Machine (SVM)^44^, Random Forest (RF)^50^, K-nearest neighbor (KNN)^51^, and Linear Discriminant Analysis (LDA)^52^. Details on the model settings can be found in the Method section.

Accuracy results obtained are reported in Table 2 and compared with those achieved exploiting the proposed MLP approach. The proposed method outperforms the traditional one by 9.48% in mean Accuracy at the single-track.

**Table 2:** Accuracy values of four classification models (SVM, RF, LDA, and KNN) built on shape and texture descriptors, compared with the results obtained using RESNET101 transfer learning descriptors.

DLD-1 versus LOX1 inhibited		
	SVM	
	Shape & texture descriptors	RESNET101
**Single-track level**	64.52 ± 6.73%	74.00 ± 4.37%
**Cluster Level**	73.49 ± 9.20%	80.48 ± 6.59%

	RF	
	Shape & texture descriptors	RESNET101
**Single-track level**	62.89 ± 4.56%	72.06 ± 5.16%
**Cluster level**	66.51 ± 10.02%	78.73 ± 5.67%

	LDA	
	Shape & texture descriptors	RESNET101
**Single-track level**	66.96 ± 11.18%	73.36 ± 6.77%
**Cluster level**	66.51 ± 12.31%	76.35 ± 8.15%

	KNN	
	Shape & texture descriptors	RESNET101
**Single-track level**	61.11 ± 7.98%	68.49 ± 6.36%
**Cluster level**	70.63 ± 6.36%	72.22 ± 5.94%

##### Relevance of the background suppression step

Background suppression is fundamental when using transfer learning procedures. In fact, if from one hand transfer learning exploitation allows achieving more generalizable and robust results, on the other hand, it may suffer from macroscopic luminance variation in the acquired videos. Being already trained on a very large dataset of highly informative images, the pretrained network may be unable to discriminate background luminance from low contrast cells. Therefore, such approach may be at risk of biasing due to the presence of macroscopic differences between different experiments. Being not yet fully addressed in the literature, we strongly believe that this aspect will represent a hot topic towards a more realistic and reliable use of transfer learning.

To demonstrate the relevance of using background suppression, we performed a sensitivity analysis by preliminarily changing the background luminance conditions applying biasing in the testing ROIs. Such procedure allows verifying the effect of such modifications on the final accuracy of classification results comparing *Background Suppression* procedure with the *No Preprocessing* condition. Table 3 summarizes the results obtained by applying a fixed increasing biasing to the original testing ROIs equal to values 0.3 (second column) corresponding to the 30% of the dynamic range, and equal to 0.5 (third column) corresponding to the 50% of the dynamic range.

**Table 3:** Accuracy classification results for No Bias condition (first column), bias value equal to 0.3 (second column), bias value equal to 0.5 (third column), using RESNET101 and SVM classifier.

RESNET101	No Bias		Bias in test = 0.3		Bias in test = 0.5	
	No Preprocessing	Background Suppression	No Preprocessing	Background Suppression	No Preprocessing	Background Suppression
**DLD-1 versus LOX1 inhibited**	79.63 ± 3.75%	74.00 ± 4.37%	78.22 ± 6.91%	74.16 ± 4.41%	56.92 ± 6.47%	74.03 ± 5.08%

It is important to observe that the exploitation of background suppression procedure allows maintaining accuracy values stable in presence of biases. On the contrary, even if the use of standard procedure (No Preprocessing) seems to increase the accuracy values in absence or in presence of small bias, it is evident that if bias increases, the performance unacceptably get worse (56.92% vs 74.03%). This fact motivates the importance of applying a preprocessing step based on background suppression.

#### Case Study 2

In this case study, we evaluated the phenotype effects of dose-dependent administering of the VEGF inhibition drug, i.e. Bevacizumab. More specifically, we consider a three class problem in which we have DLD-1 cells, DLD-1 cells treated with 125 μg/ml and DLD-1 cells treated with 250 μg/ml. This case study demonstrates the existence of the PP axis. Table 4 lists the accuracy values of classification for the three-class problem, by using the four networks as shown in Table 1.

**Table 4:** Accuracy values of classification using four deep learning networks and SVM classifier.

DLD-1 versus DLD-1 125 µg/ml versus DLD-1 250 µg/ml	ALEXNET	GOOGLENET	RESNET101	NASNETLARGE
Single-track level	76.17 ± 3.47%	76.06 ± 4.09%	77.88 ± 3.41%	73.70 ± 3.80%
Single-cluster level	86.77 ± 4.64%	84.42 ± 5.36%	84.03 ± 3.73%	81.78 ± 5.19%

**Figure 3:**
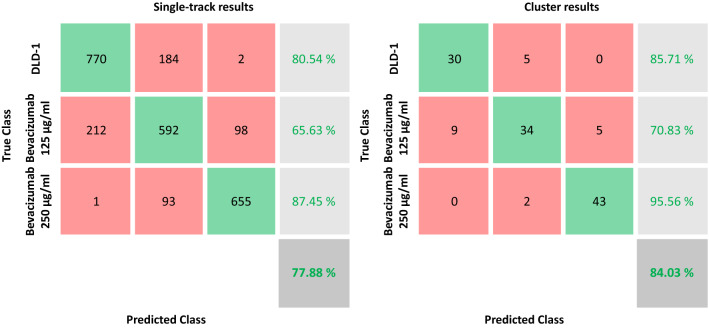
Confusion matrices of the classification task in case study 2. Single cell results (left) and cluster results (right). Third column represents in turn from top to bottom: ratio of DLD1 correctly recognized, ratio of Bevacizumab 125 μg/ml correctly detected, ratio of Bevacizumab 250 μg/ml correctly detected, unbalanced accuracy (ratio of the sum of true instances over the total number of instances).

Again the four networks show similar results, with a maximum variation in mean accuracy of 4.18%. In addition, Fig. 3 illustrates the confusion matrices for the three-class problem only for RESNET101 network and SVM classifier.

The accuracies obtained with traditional shape and texture features are compared with the performances of the proposed MLP approach (Table 5), by implementing the four classification models described above. In this case study the proposed method outperforms the traditional one by 16.17% in accuracy at the single-track level.

**Table 5:** Accuracy values of four classification models (SVM, RF, LDA, and KNN) built on shape and texture descriptors, compared with the results obtained using RESNET101 transfer learning descriptors.

DLD-1 versus DLD-1 125 μg/ml versus DLD-1 250 μg/ml		
	SVM	
	Shape & texture descriptors	RESNET101
**Single-track level**	61.71 ± 4.33%	77.88 ± 3.41%
**Cluster level**	64.46 ± 7.08%	84.03 ± 3.73%

	RF	
	Shape & texture descriptors	RESNET101
**Single-track level**	53.81 ± 4.24%	72.80 ± 4.46%
**Cluster level**	56.08 ± 8.19%	80.07 ± 6.17%

	LDA	
	Shape & texture descriptors	RESNET101
**Single-track level**	55.15 ± 8.22%	74.34 ± 4.92%
**Cluster level**	53.84 ± 12.51%	86.51 ± 6.56%

	KNN	
	Shape & texture descriptors	RESNET101
**Single-track level**	51.04 ± 6.66%	70.52 ± 4.41%
**Cluster level**	52.52 ± 11.67%	81.61 ± 5.42%

As for case study 1, we present the results achieved by applying biasing to the image of an increasing level in the pixel intensity of the ROIs in test. Table 6 lists the accuracy results for these comparative experiments. This case study confirms the necessity of the proposed preprocessing.

**Table 6:** Accuracy classification results for No Bias condition, bias value equal to 0.3 (secondo column), bias value equal to 0.5 (third column), using RESNET101 and SVM classifier.

RESNET101	No bias		Bias in test = 0.3		Bias in test = 0.5	
	No preprocessing	Background suppression	No preprocessing	Background suppression	No preprocessing	Background suppression
**DLD-1 versus DLD-1 125 µg/ml versus DLD-1 250 µg/ml**	75.55 ± 4.09%	77.88 ± 3.41%	65.29 ± 5.88%	77.90 ± 3.69%	49.23 ± 8.70%	76.70 ± 3.74%

##### Comparison with single-time point analysis

The proposed approach used a single-cell strategy during the data collection step (cell tracking, transfer learning in a crop around the cell along the track, etc.). In addition, the information extracted along the track are then combined using higher order descriptors such as standard deviation in order to capture the dynamic of the feature along the track and use this information as a signature of the cell behavior. Classification accuracy is then evaluated at *single-track level* (one track corresponds to a data) or using a majority voting of the labels assigned to all the tracks of the same cluster (*cluster level*). Such promising results have to be compared with those achieved by considering each cell time point as a data. We refer to this approach as *single-time point level* analysis. Table 7 lists the results achieved by the three approaches. It is straightforward to note that performance results collected at single-time point are very low and not comparable with those achieved by the proposed strategy.

**Table 7:** Accuracy values of the SVM classification model at single-time point level obtained using RESNET101 transfer learning descriptors and compared with those achieved by the proposed cooperative strategies (i.e., single-track and cluster level).

DLD-1 versus LOX1 inhibited	RESNET101
Single-time point level	64.15 ± 0.60%
Single-track level	74.00 ± 4.37%
Cluster level	80.48 ± 6.59%

DLD-1 versus DLD-1 125 µg/ml versus DLD-1 250 µg/ml	RESNET101
Single-time point level	64.94 ± 0.62%
Single-track level	77.88 ± 3.41%
Cluster level	84.03 ± 3.73%

## Discussion

### Comparative phenomics and proteomics results

We proposed a new fully automated platform that is able to extract significant information about the dynamic of the cell morphological phenotype. The whole process is done without the need to train any Deep Neural Network architecture and hence it is a ready to use tool for biologists and clinicians.

We showed that our method is able to discriminate phenotypic characteristics of cells related to downregulation of LOX-1 receptor (gene expression pathway) or to dose-dependent drug administration (drug effects pathway), outperforming other benchmark methods with good accuracy independently from the network chosen.

**Figure 4:**
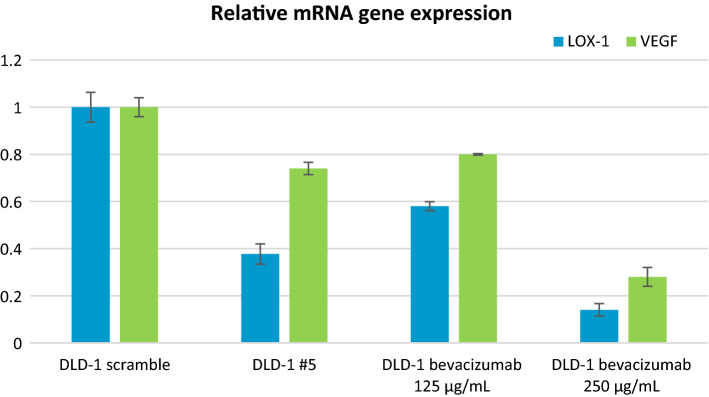
Relative gene expression of LOX-1 and VEGF-A for the four investigated classes: Control Colorectal cancer cells class (DLD-1 scramble), DLD-1 with stably down modulated LOX-1 (DLD-1#5), DLD-1 treated with Bevacizumab at 125 and 250 µg/ml.

To better emphasize the underlying molecular phenomena, we analyzed the expression of VEGF-A and of LOX-1 transcripts by Real-Time qPCR (Fig. 4).

The analysis evidenced a statistically significant reduction of LOX-1 and VEGF-A mRNA in DLD-1 cells in which LOX-1 expression was stably down modulated (DLD-1#5) compared to DLD-1scramble, in which LOX-1 is expressed (*P* < 0.05).

After treating cells with Bevacizumab (a monoclonal antibody against VEGFA) for 24 h, as expected we found that VEGF-A levels were significantly decreased at transcriptional level, especially using a concentration of 250 ug/ml. We found also downregulation of LOX-1 in cells treated with Bevacizumab. This finding suggests that a bidirectional link exists between the expression of VEGF and LOX-1.

It has been shown that a block of VEGF causes cytostasis^36^. Cytostasis is linked to change in the morphological phenotype of cells and hence to a difference in morphostatic features^37,53^.

Motility is linked to the capability of cells to dynamically change the shape of their cytoplasm to crawl. This dynamic change can be seen in the dynamics of cell’s morphology and hence in morphodynamic features.

Phenotype features gather information from both these two phenomena (cytostasis and motility), while morphostatic features (shape features) gather information only from the first.

**Figure 5:**
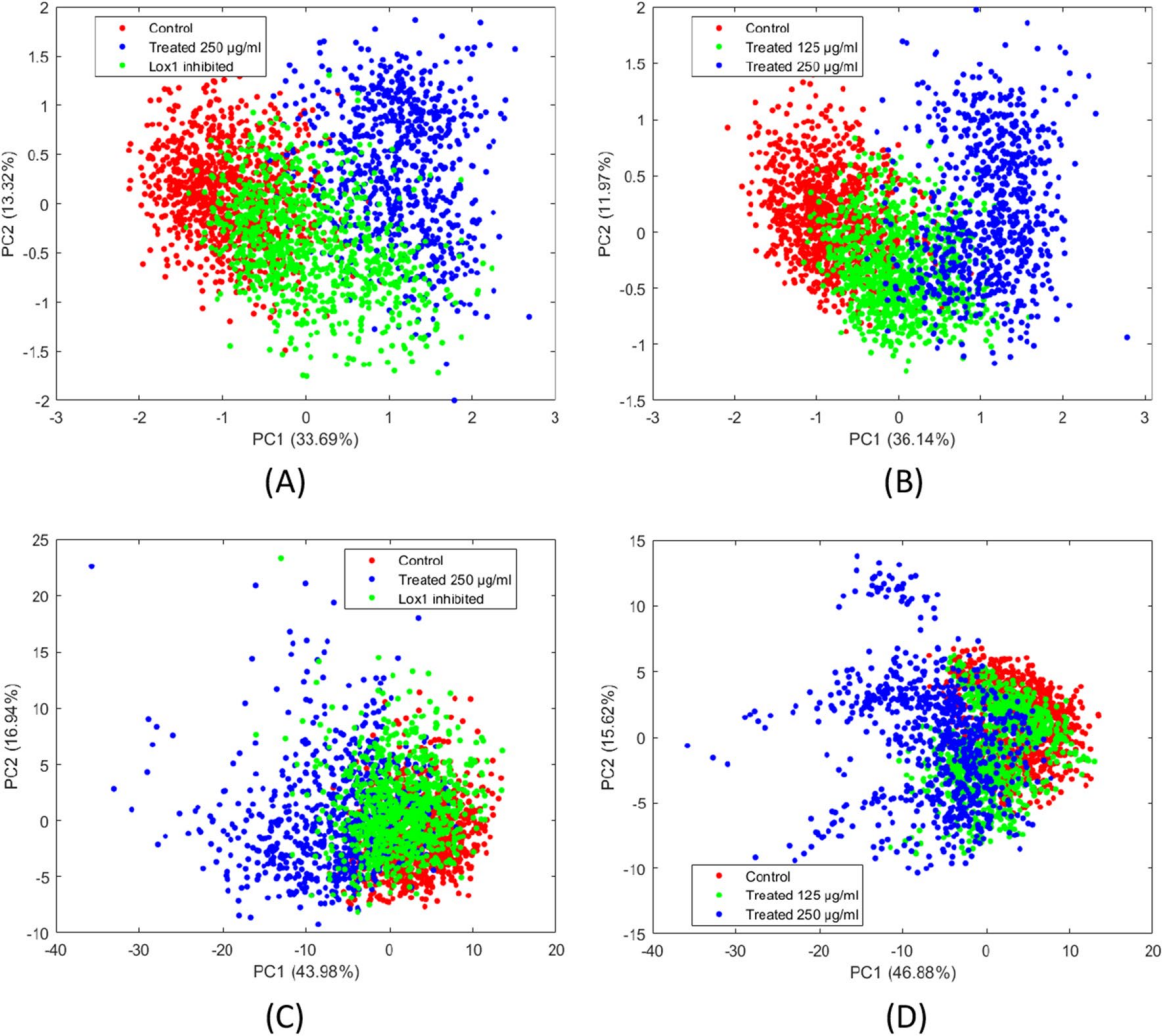
PCA Scores plot of the first two components obtained using: (**A**) Deep Features—Control DLD-1 (red) versus treated at 250 μg/ml (blue) versus LOX1 inhibited (green). (**B**) Deep Features—Control DLD-1 cells (red) versus treated at 125 μg/ml (green) versus treated at 250 μg/ml (blue). (**C**) Traditional features—Control DLD-1 (red) versus treated at 250 μg/ml (blue) versus LOX1 inhibited (green). (**D**) Traditional features—Control DLD-1 cells (red) versus treated at 125 μg/ml (green) versus treated at 250 μg/ml (blue).

As a further proof of concept of the molecular foundation of our method, we used Principal Component Analysis (PCA)^54^ to investigate the mutual relationship between the four colorectal adenocarcinoma cell classes (DLD-1 scramble, LOX-1 inhibited and treated at 125 and 250 µg/ml), with particular attention to the relation between the LOX-1 inhibited class (DLD-1 #5) and the class treated with Bevacizumab at intermediate concentration (125 µg/mL). Principal Component Analysis is an unsupervised feature extraction technique able to project data in a low dimensional space while preserving data structure. This technique is particularly useful to get intuitive visualization of data. As we can see in Fig. 5A,B LOX-1 inhibited and DLD-1 treated at 125 µg/mL are in the same region of the transformed space, as is in the LOX-1 – VEGF space. The LOX-1 inhibited class shows higher variance than those observed for Bevacizumab 125 µg/mL class as it was evidenced for the expression of VEGF and LOX-1 genes. This proves the existence of a strong link between molecular expression and cell’s phenotype dynamic suggesting its use in future precision medicine applications. Figure 5C,D illustrate analogous results achieved by using traditional shape and morphological features. The results clearly show a lower separation between the classes.

As it is shown in Fig. 5 (panels A and B), the distribution on the first two principal components related to LOX-1 inhibited is strongly overlapping the distribution related to populations treated at 125 μg/ml. This fact shows that cells with similar proteomics share also similar phenotypical traits. This proves the existence of the genomics-proteomics-phenomics axis. From methodological point of view, this indicates the fact that in the phenomics of the two experiments there are some common observational cell traits correlated to different genomics effects. Looking again at Fig. 1, this fact corroborates the non-uniqueness of the correlation among gene expression and cell phenotype that is known to be also affected by environmental interactions.

## Conclusion

In the present work, we proposed a new fully automated platform that is able to extract significant information about the dynamic of the cell morphological phenotype and to recapitulate a strict correlation among gene expression and cell phenotype. Thanks to the integration of phenomics, with image analysis, deep learning, and machine learning, we devised a more embracing approach, called Machine Learning Phenomics (MLP). The effectiveness of the proposed MLP platform has been tested against the ability to discriminate phenotype characteristics of cells related to downregulation of LOX-1 receptor (gene expression pathway) or to dose-dependent drug administration (drug effects pathway) in Colorectal Cancer Cells. The results demonstrate that the proposed platform outperformed other benchmark methods with good accuracy independently from the neural network chosen. From biological aspects, one of the potentials of the platform relies on the possibility to confirm that the inhibition of LOX-1 phenotypically emulates drug response. From a methodological point of view, this indicates the fact that in the phenomics of the two experiments there are some common observational cell traits correlated to different genomics effects corroborating the non-uniqueness of the genomics-phenomics axis that is known to be also affected by environmental interactions. The existence of a strong link between molecular and dynamic expression of the cellular phenotype opens the way for its use in future precision medicine applications.

## Methods

**Figure 6:**
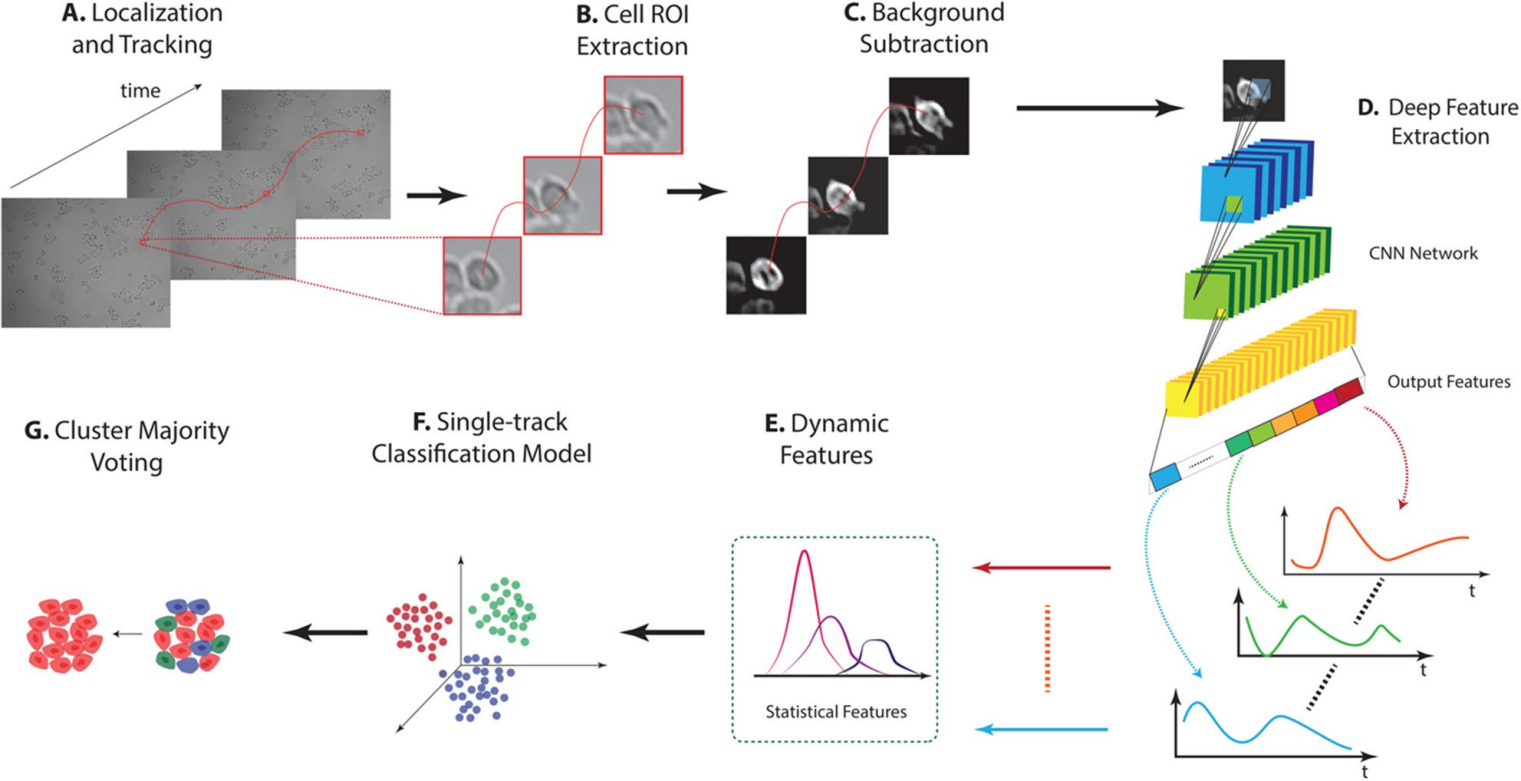
The figure depicts an overview of the method. (**A**) Cells are located and tracked. (**B**) Cell centered ROIs are extracted. (**C**) Background suppression is applied to the ROIs. (**D**) Features are extracted from the processed ROIs using a pretrained Deep Neural Network. (**E**) Starting from features signals, statistics are extracted in order to catch the dynamic of the phenomenon. (**F**) Using the statistics as features, machine learning model is constructed in order to have predictions at single cell level. (**G**) Cluster-based majority voting is finally exploited to summarize the cluster behaviour.

The first step is devoted to cell localization and tracking along the video (Fig. 6A). Then, a region of interest (ROI) containing each cell is extracted (Fig. 6B). A background subtraction procedure is then applied to each ROI to assure robustness against experimental non biological sources of variation (e.g., changes in light source intensity, changes in focus, etc.) (Fig. 6C). Using a pretrained network, the method extract features from patches (Fig. 6D). After rearrangement of the extracted features in signals, the dynamics of each feature variation is extracted via statistics extraction (Fig. 6E). The extracted statistical descriptors are then used to train a machine learning algorithm in order to infer conclusions about the biological experiments and give insight of the phenomenon at the cell (Fig. 6F) or cluster level (Fig. 6G).

### Cell-culture

Human colon cancer cell lines: DLD-1 (ATCC: CCL-221TM),) were used. mRNA expression analysis was performed on DLD-1 in which LOX-1 mRNA was stably downmodulated (LOX-1#5) as previously reported in^29^. DLD-1 scramble cells were used as control. Human colon cancer cells were grown in RPMI-1640 (Gibco, Life Technologies Corporation, Carlsbad, CA, USA) supplemented with 15% fetal bovine serum (FBS) (Euroclone, Milan, IT), Glutamine (Euroclone, Milan, IT), non-essential Amino Acids (Gibco, Life Technologies Corporation, Carlsbad, CA, USA), Penicillin–Streptomycin (Gibco, Life Technologies Corporation, Carlsbad, CA, USA).Cells were seeded at the density of 50,000 cells/cm^2^ and incubated at 37 °C, 5% CO_2_ for 24 h. Afterwards bevacizumab (Sigma-Aldrich S.r.l., Milan, Italy) was added at the concentration of 125 and 250 µg for 24 h. After this period, cells were trypsinized and collected. The experiment was performed in triplicate.

### Gene expression analysis

Total RNAs from cells were extracted by Trizol Reagent (Invitrogen Life Technologies Corporation, Carlsbad, CA, USA) following manufacturer's instructions. Treatment with DNase I-RNase-free (Ambion, Life Technologies Corporation, Foster City, CA, USA) was used to eliminate genomic DNA contamination from total RNA samples. One μg of RNA was reverse transcribed with the High-Capacity cDNA Archive kit (Life Technologies Corporation, Foster City, CA, USA) and used in RT-qPCR. LOX-1 and VEGFA mRNAs were measured by SYBR Green (Life Technologies Corporation, Foster City, CA, USA).GAPDH was used as reference gene. Primer sequences will be given upon request. The comparative ΔΔCt method was used to quantify relative gene expression levels.

### Time-lapse microscopy acquisition

A total of eight videos were acquired with a customized small-scale inverted microscope whose details are described elsewhere^43^. An ad-hoc firmware was developed in Matlab 2017a® to have full control on images acquisition and light exposure. Each video was recorded for 12 h at 1 frame per minute and a theoretical spatial resolution of 0.33 Μm/px. Two independent videos were acquired for each of the following experimental conditions:

1. DLD-1 Colorectal Cancer cells
2. DLD-1 #5
3. DLD-1 treated with Bevacizumab 125 μg/ml
4. DLD-1 treated with Bevacizumab 250 μg/ml

### Cell localization and tracking

The videos acquired are firstly processed through an already validated Matlab ® software tool called *Cell-Hunter*^55–57^. Cell-hunter is here adapted to the localization of a single population. In particular, the localization step is performed by means of Circular Hough Transform (CHT)^58,59^ with radius range of [4.3–7.6] μm. Starting from a set of localized centers, a suboptimal Assignment Problem (SOAP) problem is solved using the Munkres’ algorithm^60^. Each cell is then tracked along its movement in the video.

### Cell ROI extraction

In order to achieve a local cell-centered representation of each moving cell along the trajectory, a Region-Of-Interest (ROI) sequence is automatically extracted by cropping the video along the trajectory. Fixed dimension of 63 × 63 px (4.32e-4 mm^2^) for each crop has been selected according to the average cell radius estimated using the CHT outcome. Each ROI sequence is a signature of the cell phenotype along the video and is considered as the input of the machine learning algorithm.

### Image preprocessing

In brightfield microscopy, cells usually appear as dark objects in brighter (transparent) background due to the opacity of the cell cytoplasm and cell membrane. For further image-based processing, each ROI is inverted in order to have a brighter object over a dark background. By indicating with *I*_O_(*x*,*y*,*t*) the generic original ROI image at time *t,* assuming that graylevel belong to the normalized range [0,1], the inverted image *I*(*x*,*y*,*t*) is obtained by applying the linear map

$$I\left( {x,y,t} \right) = 1 - I_{O} \left( {x,y,t} \right)$$

Then, after computing the average luminance of $I\left( {x,y,t} \right)$, indicated with $m_{I}$, the following piecewise map is applied for background subtraction to obtain the final image $I_{BS} \left( {x,y,t} \right)$

$$I_{BS} \left( {x,y,t} \right) = \left\{ {\begin{array}{*{20}l} {0,} \hfill & {for\;I\left( {x,y,t} \right) < m_{I} } \hfill \\ {I\left( {x,y,t} \right) - m_{I} ,} \hfill & {otherwise} \hfill \\ \end{array} } \right.$$

In this way, the new ROI image $I_{BS} \left( {x,y,t} \right)$ presents an attenuated background and increased uniformity among different images and videos is achieved.

To reduce also the influence of the surrounding cells, towards an even more cell-centered analysis, a Gaussian windowing is applied to the ROI image $I_{BS} \left( {x,y,t} \right)$, resulting the new image $I_{W} \left( {x,y,t} \right)$, given by

$$I_{W} \left( {x,y,t} \right) = I_{BS} \left( {x,y,t} \right) \cdot G_{\sigma } \left( {x,y} \right)$$

where the Gaussian window $G_{\sigma } \left( {x,y} \right)$ is defined as

$$G_{\sigma } \left( {x,y} \right) = A e^{{ - \frac{{x^{2} }}{{2\sigma^{2} }}}} e^{{ - \frac{{y^{2} }}{{2\sigma^{2} }}}}$$

where *A* represents a normalization factor and $\sigma$ represents the standard deviation of the Gaussian function. In our work, $\sigma$ is set to 18 px (i.e., the average radius used in the CHT) corresponding to about 5.9 μm.

To increase the image contrast along each ROI sequence and maintain the homogeneity of the images along the sequence, we implemented the following adaptive procedure.

First, we applied an histogram stretching procedure to $I_{W} \left( {x,y,t_{0} } \right)$, where $t_{0}$ indicates the time of the first frame of the sequence, by using the following piecewise linear map

$$I_{E} \left( {x,y,t_{0} } \right) = \left\{ {\begin{array}{*{20}l} {\frac{{I_{W} \left( {x,y,t_{0} } \right) - l_{1} }}{{l_{2} - l_{1} }} ,} \hfill & {for\quad l_{1} < I_{W} \left( {x,y,t_{0} } \right) < l_{2} } \hfill \\ {0,} \hfill & {for\quad I_{W} \left( {x,y,t_{0} } \right) \le l_{1} } \hfill \\ {1,} \hfill & {for\quad I_{W} \left( {x,y,t_{0} } \right) \ge l_{2} } \hfill \\ \end{array} } \right.$$

where $I_{E} \left( {x,y,t_{0} } \right)$ represents the enhanced ROI image in the first frame, $l_{1}$ and $l_{2}$ are the 1st and the 99th quantile graylevels in $I_{W} \left( {x,y,t_{0} } \right)$.

The remaining frames $I_{E} \left( {x,y,t} \right)$ for $t > t_{0}$ are adapted to $I_{E} \left( {x,y,t_{0} } \right)$ by applying histogram matching with respect to the first frame^61^. That approach allows reducing unpredictable inconsistencies between consecutive frames that may produce biased classification results.

### Deep feature extraction

Deep Learning networks based on Convolutional Neural Networks, have been extensively used for several classification tasks in the context of biomedicine and biological applications. However, one of the most powerful approach within the context of DL is transfer learning, as highlighted in the Introduction. Transfer Learning allows transferring the network knowledge elsewhere acquired over a pre-learning step, in the case study under test. In this way, we not only solve the curse of dimensionality problem by avoiding collecting large dataset for network training, but also strongly reduce time-consuming requirements. With the aim to demonstrate the robustness of the proposed platform analysis, we compared in the same method four distinct CNNs, namely AlexNET^45^, GoogleNet^46^, ResNet101^47^, and NASNetLarge^48^. Provided that each network has its own input layer size, and pooling layer dimension, Table 8 lists all the different configurations required to apply each network. In addition, we also report the name of the deep layer used for transfer learning and the number of features extracted within.

**Table 8:** Lists of deep learning architectures selected for the test: layers used and total number of features extracted.

	AlexNET	GoogleNET	ResNET101	NasNETLarge
Layer	‘pool5’	‘pool5’	‘pool5’	‘average_pooling’
N. of features	9216	1024	2048	4032
Input Layer size	227 × 227 × 3	224 × 224 × 3	224 × 224 × 3	331 × 331 × 3

### Feature extraction and selection

As indicated in Table 8, the number of features extracted using distinct networks is largely variable. Anyway, it is well known that, especially for highly sparse images (e.g., images with many dark pixels) the extracted transferred features are sparse, or zero for the entire sequence. In fact, features activated by the deep layers, spatially correspond to specific region of the original image. Therefore, it is expected that features corresponding to regions with no pixel intensity variation (i.e., no cell activity) do not present high activation levels. To evaluate optimal features to select, each ROI sequence is transferred to a set of deep feature signals, ${f}_{i}(t)$ *(see *Fig. 6*).* To obtain a descriptor related to the dynamic evolution of the cell images, we calculated for each deep features the relative standard deviation. We use these descriptors as input of the feature selection step. In this phase, for each training set, a subset of the initial feature set will be selected based on the computation of Area Under the roC (AUC) value. More specifically, features with AUC smaller than *th*_min_ or larger than *th*_max_, where *th*_min_ = quantile(AUC,0.1) and *th*_min_ = quantile(AUC,0.9), will be selected.

### Single-cell (track) classification

After feature selection, each cell track has been considered as a unique data sample, given that the observation period is such that the temporal changes occurring in the morphological phenomics of the cells are representative of the experimental scenario. In light of this, each cell track is individually classified by four distinct classification models: Support Vector Machine (SVM)^44^, Random Forest (RF)^50^, K-nearest neighbors (KNN)^51^, and Linear Discriminant Analysis (LDA)^52^. The four classification models are used with the following settings. SVM with linear kernel function and Iterative Single Data Algorithm (ISDA) for optimization. RF with hyper parameters optimized in cross-validation (i.e., number of ensembles learning cycles, maximum number of decision splits, learning rate used for shrinkage). LDA with the assumption of diagonal covariance matrix. KNN trained on a reduced set of features (2.5%) using K equal to 9 after optimization.

All the models are trained using a half-experiment out cross-validation strategy performed by splitting up the clusters into two partitions (left clusters and right clusters).

### Cluster level classification

As extensively studied^55,62,63^, clusters of cells share common pathways during the drug administering or in response to a given insult. In addition, it is has been also observed an intrinsic heterogeneity in cells response when being in the center or at the boundary of the cluster, due to different motility constraints or different culture media composition^64^. Anyhow, it is also evidenced that cluster formation generally leads to an increased metastatic ability^65^, supporting the assumption that there exists a dominant response of the entire cluster. Therefore, provided the evidence of a concerted cell intention towards metastasis formation, we propose here to apply a consensus strategy to the categories assigned at the single-track level, in order to resume a unified cluster behavior. Majority voting is therefore implemented to assign a unique class to the entire cluster of the testing partition.
